# The effect of environment on intestinal microbial diversity of *Panthera* animals may exceed genetic relationship

**DOI:** 10.3389/fmicb.2022.938900

**Published:** 2022-07-28

**Authors:** Lei Chen, Di Xu, Mengyao Sun, Ying Li, Shen Wang, Ying Gao, Zenghao Gao, Yuying Shi

**Affiliations:** ^1^College of Life Sciences, Qufu Normal University, Qufu, China; ^2^Jinan Wildlife Park, Jinan, China

**Keywords:** *Panthera* animals, gut microbiota, genetic relationship, sampling environment, diversity analysis

## Abstract

Intestinal microbes are important symbiotes in the gastrointestinal tract of mammals, which are affected by food, environment, climate, genetics, and other factors. The gut microbiota of felines has been partially studied, but a comprehensive comparison of the gut microbiota of *Panthera* species was less reported. In this study, we compared the gut microbial composition and diversity of five species of *Panthera* (*Panthera tigris*, *Panthera leo*, *Panthera onca*, *Panthera pardus*, and *Panthera uncia*) by 16S ribosomal RNA (rRNA) amplicon sequencing. The results showed that Firmicutes was the most abundant phylum among all the *Panthera* species, followed by Actinobacteria, Fusobacteria, Bacteroidetes, Proteobacteria, Acidobacteria, Verrucomicrobia, Gemmatimonadetes, and Euryarchaeota. There were significant differences in observed species of fecal microbiota among different *Panthera* animals (*P* < 0.05), indicating that there is species specificity among *Panthera* fecal microbiota. When the samples were further grouped according to sampling locations, the comparison of the alpha diversity index between groups and beta diversity analysis showed that there were significant differences in the fecal microflora of animals from different sampling locations. Cluster analysis showed that fecal microbes of animals from the same sampling location were clustered, while gut microbes of animals of the same species, but from different sampling locations, were separated. These results indicate that environment may have more influence on mammals’ fecal microbial diversity than genetic relationships.

## Introduction

Large carnivores face greater threat and higher extinction rate than other mammals (Rabinowitz and Zeller, 2010). *Panthera* is a genus of carnivore in the family Felidae, including tiger (*Panthera tigris*), lion (*Panthera leo*), jaguar (*Panthera onca*), leopard (*Panthera pardus*), and snow leopard (*Panthera uncia*). Due to habitat degradation and loss, prey shortages, disease, and human disturbance, the population of wild *Panthera* animals declined sharply. Most of them are listed as endangered (EN) or vulnerable (VU) species by the International Union for Conservation of Nature (IUCN) (Rabinowitz and Zeller, 2010; Zhang et al., 2014; He et al., 2018; Smitz et al., 2018; Han et al., 2019).

The composition and diversity of gut microbiota are closely related to the host’s metabolism, digestion of complex macromolecules, and pathogen defense. They can decompose indigestible nutrients and provide energy for the host (Erwin et al., 2017; Li et al., 2018), which are of great significance to the survival and environmental adaptation of wild animals (Hale et al., 2017). On the other hand, gut microbes are an important indicator to measure the health status of wild animals. Changes in gut microbes can also be used as an important reference to evaluate the habitat degradation of wild animals (Han et al., 2019). Many factors can influence the composition and diversity of gut microbiota, such as diet, age, gender, environment, and genetics (Dominianni et al., 2015; Guo et al., 2019; Grieneisen et al., 2021). At present, the basic composition of gut microbes of *Panthera* animals has been preliminarily studied. Proteobacteria, Firmicutes, Actinobacteria, and Fusobacteria are the dominant phyla in the intestinal tracts of *Panthera* animals (Zhang et al., 2014; He et al., 2018; Han et al., 2019), which are generally consistent with most mammals (Li et al., 2018; Yang et al., 2018; Gao et al., 2019). It is believed that animals in *Panthera* rely on a high-protein and high-fat diet as their main energy source and the high proportion of Proteobacteria and Firmicutes in their intestinal is closely related to their diet (Rabinowitz and Zeller, 2010; Zhang et al., 2014; Han et al., 2019). Mittal et al. (2020) compared the composition of the gut microbiota of 3 *Panthera* species (tiger, leopard, and lion) from India and found that diet and geographical location play a crucial role in shaping the gut microbiota (Mittal et al., 2020). These studies provided basic data for the study of the gut microbial diversity of *Panthera*. In the present study, we collected fecal samples of 5 species of *Panthera* (*Panthera tigris*, *Panthera leo*, *Panthera onca*, *Panthera pardus*, and *Panthera uncia*) from three regions of China (Jinan, Linyi, and Weihai) and used 16S ribosomal RNA (rRNA) amplicon sequencing to search for the core flora of *Panthera*’s fecal microbiota. Then, we compared the composition and diversity of *Panthera*’s fecal microbiota among species and among sampling locations to discuss the influence of the genetic relationship and environment on the fecal microbiota. The result of this study will provide a reference to explore the influencing factors of *Panthera*’s gut microbial diversity and provide basic data for *Panthera*’s nutrition and health research, as well as environmental adaptation research.

## Materials and methods

### Samples collection

Fecal samples were collected from 24 animals of 5 species in *Panthera* from Ji’nan (JN) Wildlife Park, Weihai (WH) XiXiakou Wildlife Park, and Linyi (LY) Wildlife Park in Shandong Province, China (Table 1). The experimental animals did not suffer from any diseases and had not been fed or treated with any drugs, antibiotics, and intestinal probiotics for 3 months before sampling. Fresh fecal samples were collected aseptically immediately after defecation, transported to the laboratory on dry ice within 24 h, and stored at −80°C until DNA was extracted. No harm was done to the animals during the sampling period and no habitat was destroyed. This study was conducted under the regulations of the Bioethics Committee of Qufu Normal University and complied with the regulations of the China Wildlife Conservation Association and the relevant requirements of Chinese laws.

**TABLE 1:** Sample information and grouping.

Sample	Species name	Sampling location	Group 1 (species)	Group 2 (location)	Sample	Species name	Sampling location	Group 1 (species)	Group 2 (location)
leo1M	*Panthera leo*	Ji’nan	P_leo	JN	pardus1M	*Panthera pardus*	Ji’nan	P_pardus	JN
leo2M	*Panthera leo*	Ji’nan	P_leo	JN	pardus2M	*Panthera pardus*	Ji’nan	P_pardus	JN
leo3F	*Panthera leo*	Ji’nan	P_leo	JN	pardus3M	*Panthera pardus*	Weihai	P_pardus	WH
leo4F	*Panthera leo*	Ji’nan	P_leo	JN	pardus4M	*Panthera pardus*	Weihai	P_pardus	WH
tigris1M	*Panthera tigris*	Ji’nan	P_tigris	JN	pardus5F	*Panthera pardus*	Ji’nan	P_pardus	JN
tigris2M	*Panthera tigris*	Ji’nan	P_tigris	JN	pardus6F	*Panthera pardus*	Weihai	P_pardus	WH
tigris3F	*Panthera tigris*	Ji’nan	P_tigris	JN	pardus7F	*Panthera pardus*	Linyi	P_pardus	LY
tigris4F	*Panthera tigris*	Ji’nan	P_tigris	JN	pardus8F	*Panthera pardus*	Linyi	P_pardus	LY
onca1M	*Panthera onca*	Ji’nan	P_onca	JN	pardus9F	*Panthera pardus*	Linyi	P_pardus	LY
onca2M	*Panthera onca*	Weihai	P_onca	WH	pardusXM	*Panthera pardus*	Linyi	P_pardus	LY
onca3M	*Panthera onca*	Weihai	P_onca	WH	uncia1M	*Panthera uncia*	Ji’nan	P_uncia	JN
onca4M	*Panthera onca*	Weihai	P_onca	WH	uncia2F	*Panthera uncia*	Ji’nan	P_uncia	JN

### Genomic DNA extraction and PCR amplification

Genomic DNA was extracted from fecal samples by cetyltrimethylammonium bromide (CTAB) method. We used the Thermo Scientific™ NanoDrop™ One and the Invitrogen Qubit 4.0 to measure the concentration and purity of genomic DNA. An appropriate amount of genomic DNA was taken and diluted to 1 ng/μl in sterile water. Then, the diluted genomic DNA was used as a template. 16S rRNA gene V4 region-specific primers (515F and 806R) with Barcode, Phusion^®^ High-fidelity PCR Master Mix with GC Buffer (New England Biolabs), and high-fidelity enzyme were used for PCR amplification. The PCR products were equally mixed with 1× loading buffer and detected by 2% agarose gel electrophoresis. The TruSeq^®^ DNA PCR-Free Sample Preparation Kit (Illumina, United States) was used to construct the library. After the library was quantified by the Qubit and quantitative PCR (Q-PCR) detection, the HiSeq 2500 was used for sequencing with the read length of 2 bp × 250 bp.

### Data analysis

Data from each sample were separated according to the barcode sequence and PCR primer sequence. FLASH was used to splice the reads of each sample after the barcode and primer sequences were cut off (Magoc and Salzberg, 2011). After rigorous filtering of the splicing sequences (raw tags), high-quality clean tags were obtained (Bokulich et al., 2013) and were compared with the species annotation database for detection and removal of chimeric sequences to obtain the final effective tags (Rognes et al., 2016).

### Operational taxonomic units clustering and annotation

Uparse software was used to cluster operational taxonomic units (OTUs) with 97% identity for all the effective tags of all the samples (Haas et al., 2011). According to the clustering results, a Venn diagram was drawn to analyze the common and unique OTUs among 5 species of *Panthera*. By comparing with small subunit rRNA (SSU rRNA) database for species annotation analysis (set threshold of 0.8–1), the community composition of each sample at different classification levels was obtained (Wang et al., 2007). Then, MUSCLE (version 3.8.31) was used for fast multisequence alignment to obtain the phylogenetic relationships of all the OTUs representative sequences (Quast et al., 2012).

### Alpha diversity and beta diversity analyses

We plotted the rarefaction curve, rank clustering curve, and species accumulation curve to determine whether the sequencing depth could truly reflect the microbial community diversity in the samples. Differences in species richness and diversity of microbial communities in each sample were assessed by calculating the alpha diversity indices [Shannon index, Simpson index, Chao1, abundance-based coverage estimator (ACE), goods_coverage, and PD_whole_tree] under the 97% identity threshold.

To compare the influence of genetic relationship and environment on the gut microbiota of *Panthera* animals, we grouped fecal samples according to species and sampling locations (Table 1). Beta analysis by using R software (version 2.15.3) was calculated to compare the composition of microbial communities in the different groups. principal component analysis (PCA), principal co-ordinates analysis (PCoA), and non-metric multi-dimensional scaling (NMDS) analyses were performed by using R software (version 2.15.3) to compare the similarity and divergence of gut microbiome between groups. Quantitative insights into microbial ecology (QIIME) software (version 1.9.1) was used to calculate weighted and unweighted UniFrac distance and build the unweighted pair-group method with arithmetic mean (UPGMA) cluster tree (Caporaso et al., 2010).

### Statistical test

To compare whether the differences between groups were significant, we performed the Wilcoxon signed-rank test on the alpha diversity indices by using the R agricolae software package (Dexter, 2013). analysis of similarities (ANOSIM) and multi response permutation procedure (MRPP) analyses were performed by using the R vegan software package to assess whether the differences between groups were significantly greater than the differences within groups. To find the species that contributed significantly to the difference between groups, LDA effect size (LEfSe) software was used for LEfSe analysis and the screening value of the linear discriminant analysis (LDA) score was set as 4.

### Function prediction

Tax4Fun R program package was used to predict the gut microbial function of *Panthera* animals. The Tax4Fun function was predicted by the nearest neighbor method based on the minimum sequence similarity. The 16S rRNA gene sequences of prokaryotes from the kyoto encyclopedia of genes and genomes (KEGG) database were extracted and compared with the SILVA SSU Ref NR database basic local alignment search tool (BLAST bitscore > 1,500) by using the BLASTN algorithm to establish a matrix. Then, OTUs were clustered with the SILVA database sequence as the reference sequence to obtain functional annotation information. According to the annotation results, the top 10 functions with maximum abundance in each sample or group at each annotation level were selected to generate a histogram of relative abundance of functions, to visually view the functions with a high relative abundance and their proportion at different annotation levels of each sample. We plotted a Venn diagram to show the abundance of common and unique functions among groups and compared the gene function distribution among groups by the Wilcoxon signed-rank test using R agricolae software package. According to the functional annotation and abundance information of the samples in the database, the top 35 functions and abundance information in each sample were selected to plot a heat map. PCA analysis was performed to evaluate the functional similarity of genes among groups and UniFrac cluster analysis was performed based on the functional differences.

## Results

### Operational taxonomic unit analysis and annotation

A total of 144,174 raw sequences were obtained by the Illumina HiSeq sequencing. After splicing, quality control, and removal of chimeric filtering, 10,561 effective tags were obtained on average for subsequent analysis. By the OTUs clustering and annotation, a total of 873 OTUs were obtained. The five *Panthera* species shared 91 OTUs. There were 14 unique OTUs in the gut microbes of tigers, 9 unique OTUs in the gut microbes of lions, 4 unique OTUs in the gut microbes of jaguars, 3 unique OTUs in the gut microbes of snow leopards, and 303 unique OTUs in the gut microbes of leopards (Supplementary Figure 1A). Among the 303 unique OTUs in leopards, the taxa in Firmicutes were the most abundant. On the other hand, fecal microbes of *Panthera* animals from three sampling locations shared 166 OTUs. There were 100 unique OTUs in the JN group, 238 unique OTUs in the LY group, and 182 unique OTUs in the WH group (Supplementary Figure 1B).

According to the results of species annotation, 24 phyla, 38 classes, 69 orders, 114 families, and 248 genera were annotated. At the phylum level, Firmicutes was the most abundant bacterial phylum in the fecal sample of *Panthera* animals, followed by Actinobacteria, Fusobacteria, Bacteroidetes, Proteobacteria, Acidobacteria, Verrucomicrobia, Gemmatimonadetes, and Euryarchaeota. Acidobacteria are absented from the gut microbial community of group P_tigris. Acidobacteria did not exist in most samples of the gut microbial community of group P_leo and group P_uncia, except P_ leo_3F and P_uncia_2F. There was no Verrucomicrobia in group P_tigris, group P_leo, and group P_uncia. In addition, Gemmatimonadetes was also not found in the gut microbiota of group P_leo and group P_uncia. In regional grouping, Acidobacteriota and Verrucomicrobiota were only found in WH (Supplementary Figure 2).

Coriobacteriaceae was the most abundant bacterial family, followed by Peptostreptococcaceae, Erysipelotrichaceae, Fusobacteriaceae, Lachnospiraceae, Campylobacteraceae, Bacteroidaceae, Enterobacteriaceae, and Veillonellaceae. However, Campylobacteraceae was not found in group P_tigris’ fecal microbiota and the proportion of Campylobacteraceae in group P_leo was less than 0.01% (Supplementary Figure 3). At the genus level, *Collinsella*, *Peptoclostridium*, *Solobacterium*, *Fusobacterium*, *Campylobacter*, *Bacteroides*, *Paeniclostridium*, and *Megamonas* were more abundant in *Panthera* animals’ gut microbiota (Supplementary Figure 4).

### Alpha diversity analysis

The results of the alpha diversity index showed that the observed species of leopards was the highest among the five species (Table 2). The rarefaction curve and rank abundance curve also showed that leopards had the highest number of OTUs at the same sequencing depth (Supplementary Figure 5). The results of alpha diversity analysis showed that the Shannon and Simpson indices of jaguars were the highest. In regional grouping, both the rarefaction curve and the rank abundance curve showed that the number of species annotated in the gut microbial community of the JN group was the least at the same sequencing depth (Supplementary Figure 6).

**TABLE 2:** Alpha diversity indices of *Panthera* animals’ intestinal microbiota (mean value).

	Observed species	Shannon	Simpson	Chao1	ACE	Goods coverage	PD whole tree
P_leo	136	3.938	0.899	150.558	159.719	1	14.962
P_tigris	169	3.805	0.882	182.552	187.284	1	17.511
P_onca	221	4.659	0.931	256.572	256.741	0.999	32.326
P_pardus	245	4.591	0.898	273.118	275.372	0.999	29.406
P_uncia	131	3.766	0.887	166.908	158.375	1	17.529

### Comparative analysis of multiple samples

**FIGURE 1:**
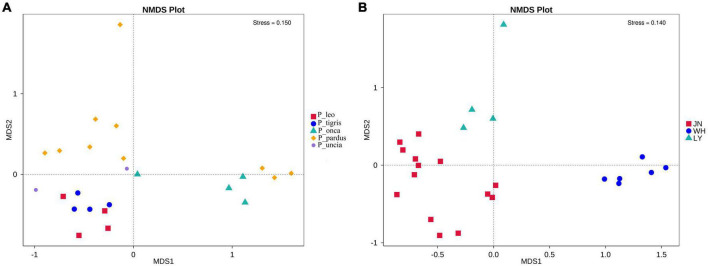
NMDS analysis of fecal microbiota among species groups **(A)** and sampling location groups **(B)**.

**FIGURE 2:**
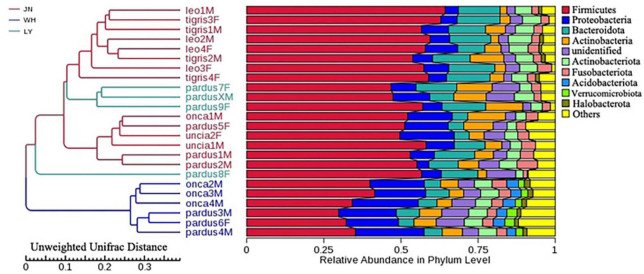
The UPGMA clustering analysis of fecal microbiota based on unweighted UniFrac distance.

The results of NMDS analysis showed that group P_tigris and group P_leo clustered together, while group P_onca, group P_uncia, and group P_pardus gathered together (Figure 1A). This was also confirmed by the UPGMA cluster analysis based on weighted and unweighted UniFrac distance, suggesting a correlation between gut microbial composition and genetic relationships in *Panthera* animals (Supplementary Figure 7). Second, when we regrouped all the samples according to the sampling locations, PCA and NDMS results showed that samples from the same sampling location clustered together (Figure 1B). The UPGMA clustering tree based on weighted and unweighted UniFrac distance showed that all the samples were obviously divided into two branches, samples from Weihai (WH group) clustered together, while samples in groups JN and LY clustered into the other branch (Figure 2 and Supplementary Figure 8).

### Differential analysis of gut microbial communities

#### Difference analysis between groups

The results of ANOSIM analysis based on the Bray–Curtis distance showed that the difference between groups was greater than the difference within groups (*R* > 0), which proved that the grouping by species and by sampling sites was statistically significant (Supplementary Table 1). The Wilcoxon signed-rank test results of the alpha diversity index showed that there was no significant difference in the Shannon indices, Simpson indices, and goods coverage indices among the five species (*P* > 0.05). However, there were significant differences in observed species between group P_pardus and group P._leo and between group P_leo and group P_onca (*P* < 0.05). Chao1, ACE, and PD whole-tree indices also showed significant differences among different *Panthera* species (Table 3). Significant differences were also found between group P_pardus and group P_leo, group P_onca and group P_leo, group P_pardus, and group P_tigris, and group P_onca and group P_tigris by MRPP analysis (Supplementary Table 2). In addition, analysis of molecular variance (AMOVA) analysis based on unweighted UniFrac distance also showed significant differences among P_leo, P_onca, P_pardus, P_tigris, and P_uncia (unweighted UniFrac *P* = 0.002; weighted UniFrac *P* = 0.05) (Supplementary Table 3). These results indicated that there was species specificity in the gut microbes of the five *Panthera* species.

**TABLE 3:** The Wilcoxon signed-rank test of the alpha diversity indices among species groups and among location groups (*P*-values, *P* < 0.05 indicates significant difference and *P* < 0.01 indicates extremely significant difference).

	Observed species	Shannon	Simpson	Chao1	ACE	Goods coverage	PD whole tree
P_leo vs. P_onca	0.029	0.087	0.266	0.017	0.012	0.11	0.007
P_leo vs. P_pardus	0.018	0.179	0.512	0.009	0.010	0.127	0.009
P_leo vs. P_tigris	0.602	0.958	0.624	0.483	0.512	1.000	0.414
P_onca vs. P_pardus	0.813	0.456	0.490	0.833	0.627	0.694	0.450
P_onca vs. P_tigris	0.083	0.079	0.116	0.071	0.047	0.111	0.040
P_pardus vs. P_tigris	0.067	0.161	0.221	0.053	0.055	0.127	0.077
JN vs. LY	0.001	1.000	0.368	0.001	0.001	0.066	0.006
JN vs. WH	0.000	0.002	0.005	0.000	0.000	0.148	0.000
LY vs. WH	0.469	0.014	0.004	0.418	0.469	0.576	0.144

In regional grouping, the Wilcoxon signed-rank test showed that there was an extremely significant difference in Shannon indices between the groups JN and WH (*P* < 0.01) and a significant difference between the groups LY and WH (*P* < 0.05). There were extremely significant differences in Simpson indices between the groups JN and WH and between the groups LY and WH (*P* < 0.01). There were also extremely significant differences in observed species indices between the groups JN and WH and between the groups JN and LY (*P* < 0.01) (Table 3). At the same time, the results of MRPP analysis based on the Bray–Curtis distance (Supplementary Table 4) and AMOVA analysis (Supplementary Table 3) also showed that there were significant differences in the fecal microbiota of *Panthera* animals among the groups JN, WH, and LY (*P* < 0.05). These results showed that sampling location had a significant effect on the fecal microbiota of *Panthera* animals.

#### Analysis of different species between groups

**FIGURE 3:**
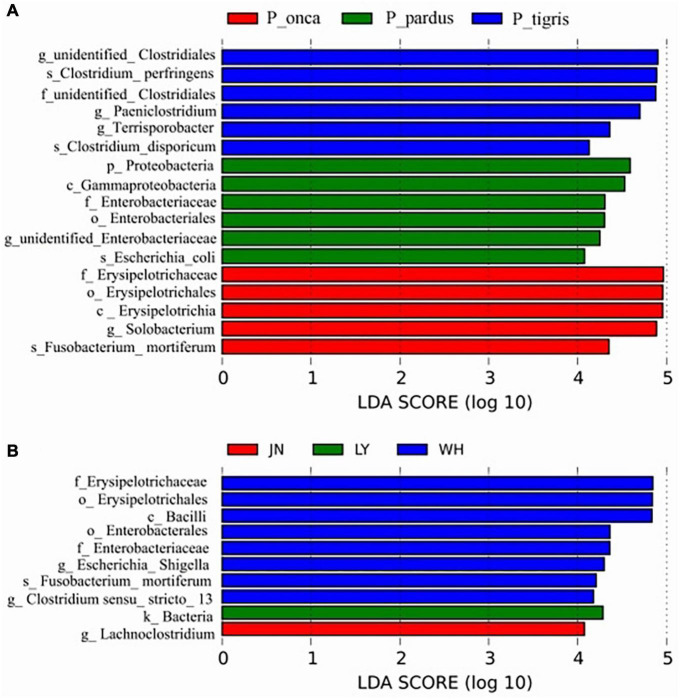
LDA value distribution histogram shows the representative biomarkers in different species groups **(A)** and different sampling location groups **(B)**.

The LEfSe analysis results showed that the differences among species mainly existed in the phyla Firmicutes and Proteobacteria. The representative biomarkers in Firmicutes were the class Erysipelotrichia and order Erysipelotrichales. The representative biomarkers in phylum Proteobacteria were the class Gammaproteobacteria and order Enterobacteriales. The differences among samples from different sampling locations mainly existed in the phyla Firmicutes, Fusobacteriota, and Proteobacteria. The most representative biomarkers in Firmicutes were the family Erysipelotrichaceae in the class Bacilli and the family Clostridiacea and Lachnospiraceae in the class Clostridia. The representative biomarkers in Fusobacteriota were the species *Fusobacterium_mortiferum* in the genus *Fusobacterium*, family Fusobacteriaceae. The representative biomarkers of Proteobacteria were the class Gammaproteobacteria, order Enterobacterales, family Enterobacteriaceae, and genus *Escherichia_Shigella* (Figure 3).

### Function prediction

**FIGURE 4:**
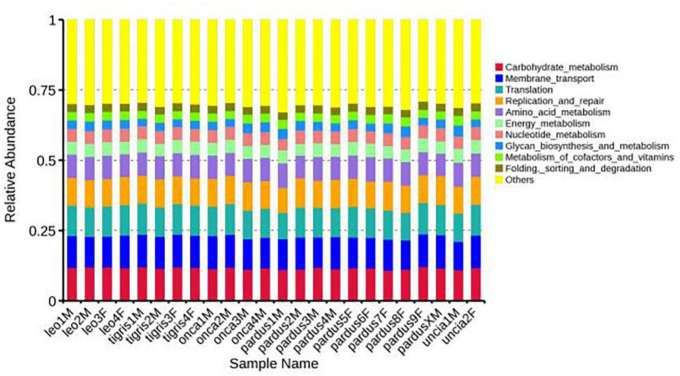
Relative functional abundances of fecal microbes annotated at the KEGG level 2.

According to the annotation results of the KEGG database, the predicted functions of *Panthera* fecal microbes at level 1 mainly included metabolism, genetic information processing, and environmental information processing. At level 2, the functions mainly included carbohydrate metabolism, membrane transport, and translation. The functions at level 3 mainly included transporters, DNA repair and recombination proteins, transfer RNA biogenesis, etc. (Figure 4 and Supplementary Figures 9, 10).

We plotted a Venn diagram to analyze the common and specific information of genes among different groups; the results showed that the 5 species shared 6,034 annotation functions, while samples from 3 locations shared 6,267 annotation functions (Supplementary Figure 11). In addition, based on the database annotation results, the differences in annotation function between groups were analyzed by the Wilcoxon signed-rank test. At level 2, there were significant differences in signal transduction between group P_leo and group P_onca and between group P_leo and group P_pardus. There were significant differences in glycan biosynthesis and metabolism between group P_leo and group P_tigris and between group P_pardus and group P_tigris (Supplementary Table 5). There was a significant difference in cellular community-prokaryotes between the groups JN and WH.

Heat maps based on the functional abundances of samples annotated in the database showed that among different species, the abundance of cellular processes and organic systems in group P_uncia was the highest and the abundance of genetic information processing and environmental information processing in group P_tigris was the highest. Besides, cellular processes and genetic information processing had the highest abundance in the LY group, organic systems and environmental information processing had the highest abundance in the JN group, while the most abundant function in the WH group was metabolism (Supplementary Figure 12).

## Discussion

A comparative analysis of the gut microbiota of three species of *Panthera* found that Firmicutes was the most abundant bacterial phylum in tiger and leopard, while the most abundant bacterial phylum in lion gut microbiota was Fusobacteria (Mittal et al., 2020). In this study, Firmicutes was found to be the most abundant bacterial phylum in the fecal flora of all of the five *Panthera* species and we believe that this is closely related to the high-protein diet of *Panthera* species (Sugden et al., 2020). In addition, in the present study, Acidobacteria was found to be one of the top ten bacterial phyla in the gut microbiota of *Panthera* animals, except group P_tigris. Verrucomicrobia was also the top ten abundant bacterial phyla in group P_onca and group P_pardus, but was not found in group P_leo, group P_tigris, and group P_uncia. As previously reported, Acidobacteria and Verrucomicrobia were not found in the fecal microbiota of lions (Mittal et al., 2020), tigers (He et al., 2018; Ning et al., 2020), and snow leopards (Zhang et al., 2014). Both Acidobacteria and Verrucomicrobia contain enzymes that encode cellulose and hemicellulose and are involved in plant degradation (Kanokratana et al., 2011; Chen et al., 2017; Karmacharya et al., 2019). We speculate that a certain proportion of Acidobacteria and Verrucomicrobia in *Panthera* animals’ fecal samples may be related to their prey (Grieneisen et al., 2019; Mittal et al., 2020).

In this study, we found significant differences in the gut microbial communities of *Panthera* across sampling sites and species. However, the results of the UPGMA clustering tree showed that the microbiota of the samples from phylogenetically closely related species did not cluster together. The microbiota of samples from the same sampling locations clustered. These results indicate that the environment of the sampling sites might have a higher influence on the gut microbial communities of *Panthera* than genetic relationships (Moeller et al., 2013; Grieneisen et al., 2021). Mittal et al. (2020) indicated that habitat plays a key role in shaping gut microbial composition rather than the influence of host genetic relationships. Previous studies have found that climatic conditions of the habitat affect the composition of animals’ gut microbiota (Wu et al., 2017). Rainfall is considered to be an important ecological factor affecting gut microbes in mammals. Studies found that cumulative rainfall was significantly associated with the relative abundance of 63% of the bacterial families in the geladas (*Theropithecus gelada*) gut (Baniel et al., 2021). Some other studies found that the soil characteristics (pH, sodium content) of animal habitats are also important factors affecting gut microbes. For example, the number of baboon gut microbial OTUs was found to be lower in the habitat where the soil has a higher sodium content (Grieneisen et al., 2019). In our study, the number of OTUs in the WH was lower than that in the JN and LY. The samples of the group WH came from the coastal city Weihai. Due to the influence of the ocean, the air humidity and the sodium content in the soil in Weihai are higher than that of other two sampling sites (Ji’nan and Linyi). Differences in climatic conditions and soil sodium content may account for differences in species composition and diversity of fecal microbiota among JN, LY, and WH.

In this study, we compared the composition and diversity of the gut microbial communities of *Panthera* animals by 16S rRNA amplicon sequencing and found species-specific differences among the gut microbiota of *Panthera*. However, cluster analysis showed that the environment of the sampling site may have a higher influence on the gut microbiota than the genetic relationship. Greater environmental effects on the gut microbiome than genetic effects have been suggested in several studies (Grieneisen et al., 2021). For example, chimpanzees and gorillas in the same region share more bacterial communities than the same species in different regions (Moeller et al., 2013). Our study further demonstrates that the environment has a significant effect on the gut microbiota of *Panthera* and even more than the influence of the host genetic relationship. In the future, we will study the function of the *Panthera* animals’ gut microbiota by using techniques such as metagenomics and metabolomics to scientifically explain the difference in the gut microbiota of *Panthera* animals, to provide more effective data for understanding the environmental effects on the gut microbiota, and provide a reference for understanding the functional contribution of gut microbes in animal survival, health, and environmental adaptation.

## Data availability statement

The raw data presented in this study can be found in National Center for Biotechnology Information (NCBI) Sequence Read Archive (SRA) data base through the accession number PRJNA832542 (SRA numbers SRX15117472 – SRX15117495 and PRJNA832542). The names of the repository/repositories and accession number(s) can be found in the article/Supplementary material.

## Ethics statement

The animal study was reviewed and approved by Bioethics Committee of Qufu Normal University.

## Author contributions

LC conceived, designed, and performed the experiments. DX analyzed the data and wrote the manuscript. MS, ZG, SW, and YS modified the manuscript. YG and YL collected the samples. All authors have read and approved the final version of the manuscript.

## Supplementary material

Supplementary Figure 1 A venn diagram shows the amount of shared and unique fecal microbes among species groups **(A)** and sampling location groups **(B)**.

Supplementary Figure 2 The relative species abundance of fecal microbes at the phylum level.

Supplementary Figure 3 The relative abundance of fecal microbes at the family level.

Supplementary Figure 4 The relative abundance of fecal microbes at the genus level.

Supplementary Figure 5 **(A)** Rarefaction curve of the fecal microbiota of species groups. **(B)** Rank abundance curve of the fecal microbiota of species groups.

Supplementary Figure 6 **(A)** Rarefaction curve of the fecal microbiota of sampling location groups. **(B)** Rank abundance curve of the fecal microbiota of sampling location groups.

Supplementary Figure 7 The UPGMA clustering analysis of different species is based on weighted **(A)** and unweighted **(B)** UniFrac distance.

Supplementary Figure 8 The UPGMA clustering analysis of different sampling location groups based on weighted UniFrac distance.

Supplementary Figure 9 Relative functional abundances of fecal microbes annotated at the KEGG level 1.

Supplementary Figure 10 Relative functional abundances of fecal microbes annotated at the KEGG level 3.

Supplementary Figure 11 A venn diagram shows the abundance of functions shared and unique among different species **(A)** and different sampling location groups **(B)**.

Supplementary Figure 12 The clustering heat map shows the difference in predicted microbial functions among different groups.

Supplementary Table 1 ANOSIM analysis based on Bray–Curtis distance (*R-*value).

Supplementary Table 2 MRPP analysis of OTU abundance of different species.

Supplementary Table 3 AMOVA analysis of different groups (*P-*value, *P* < 0.05 indicates significant difference, *P* < 0.01 indicates extremely significant difference).

Supplementary Table 4 MRPP analysis of OTU abundance of different sampling groups.

Supplementary Table 5 Functions with significant different abundance at Level 2 of different species groups.
