# Supplementary figures and images for: The effect of environment on intestinal microbial diversity of Panthera animals may exceed genetic relationship
Source: Front Microbiol. 2022 Jul 28;13:938900. doi: 10.3389/fmicb.2022.938900 (PMC9366613; doi:10.3389/fmicb.2022.938900)

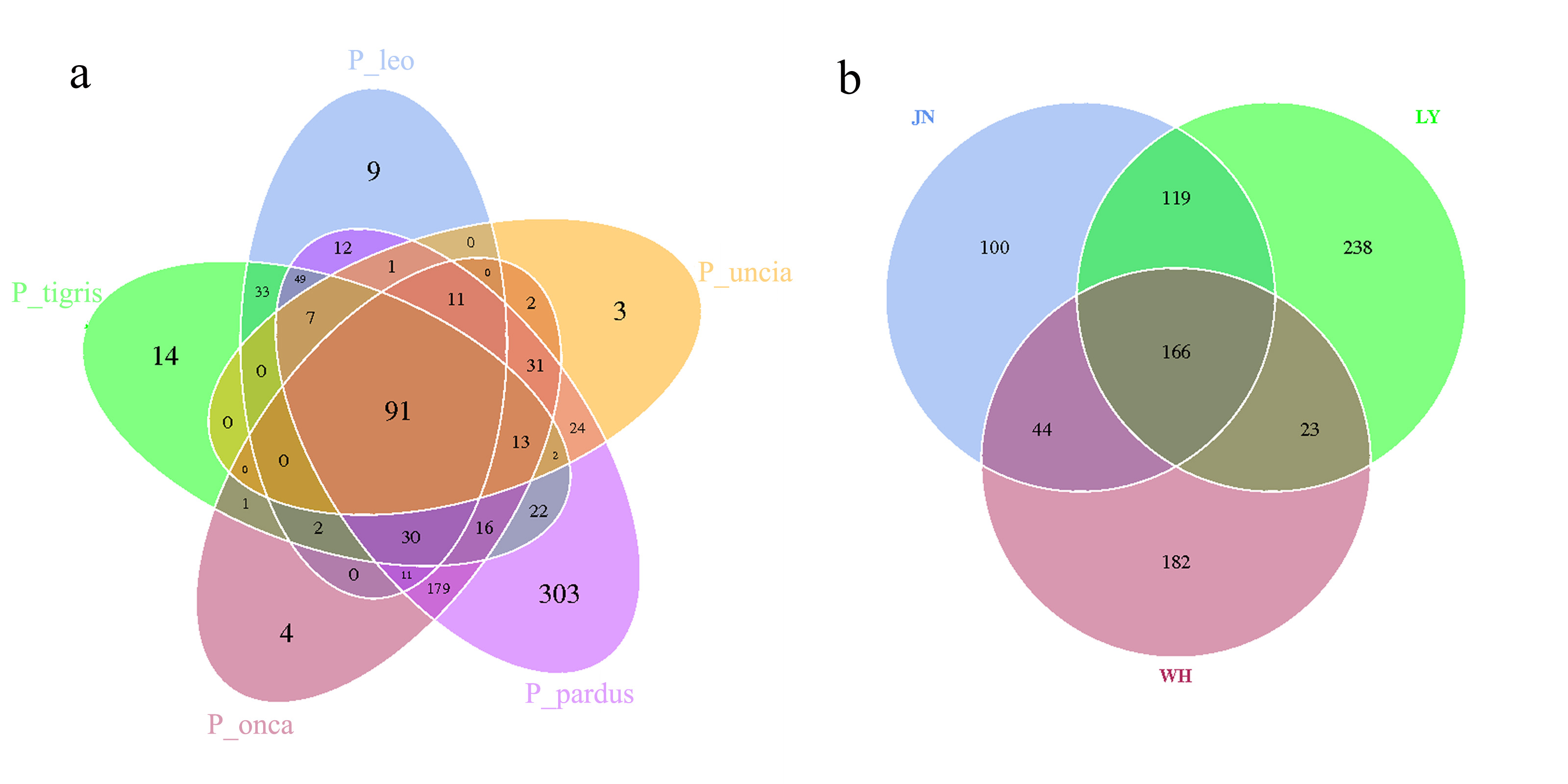

Supplement: Supplementary Figure 1 — A venn diagram shows the amount of shared and unique fecal microbes among species groups (A) and sampling location groups (B). [file Image_1.JPEG]

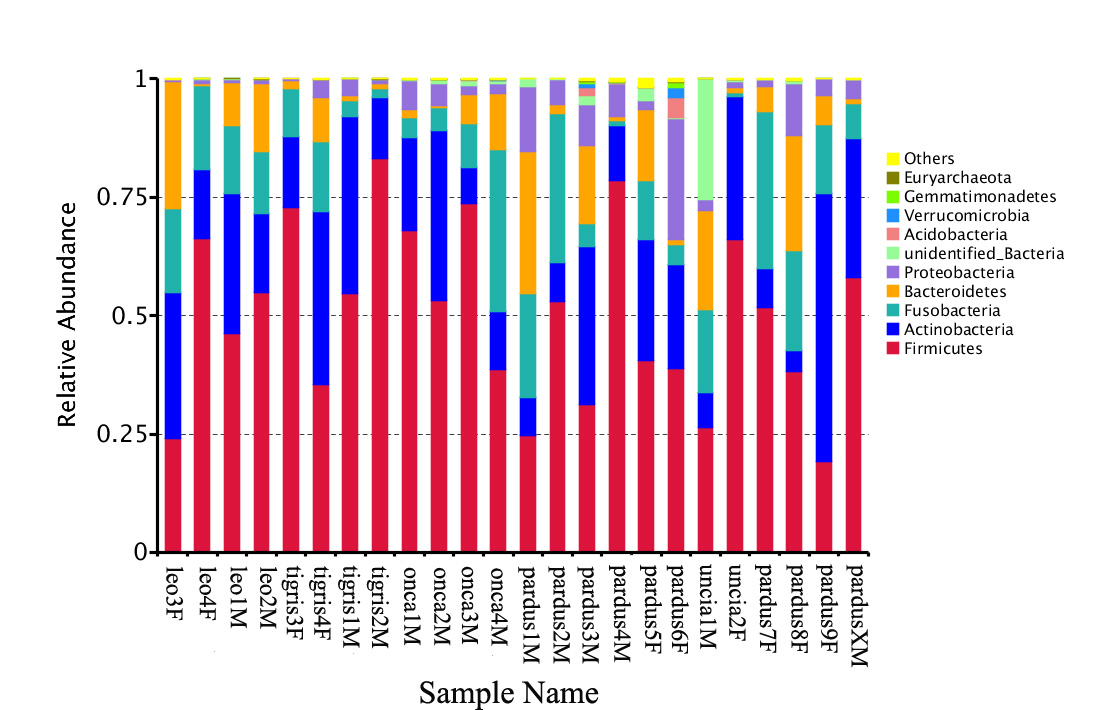

Supplement: Supplementary Figure 2 — The relative species abundance of fecal microbes at the phylum level. [file Image_2.JPEG]

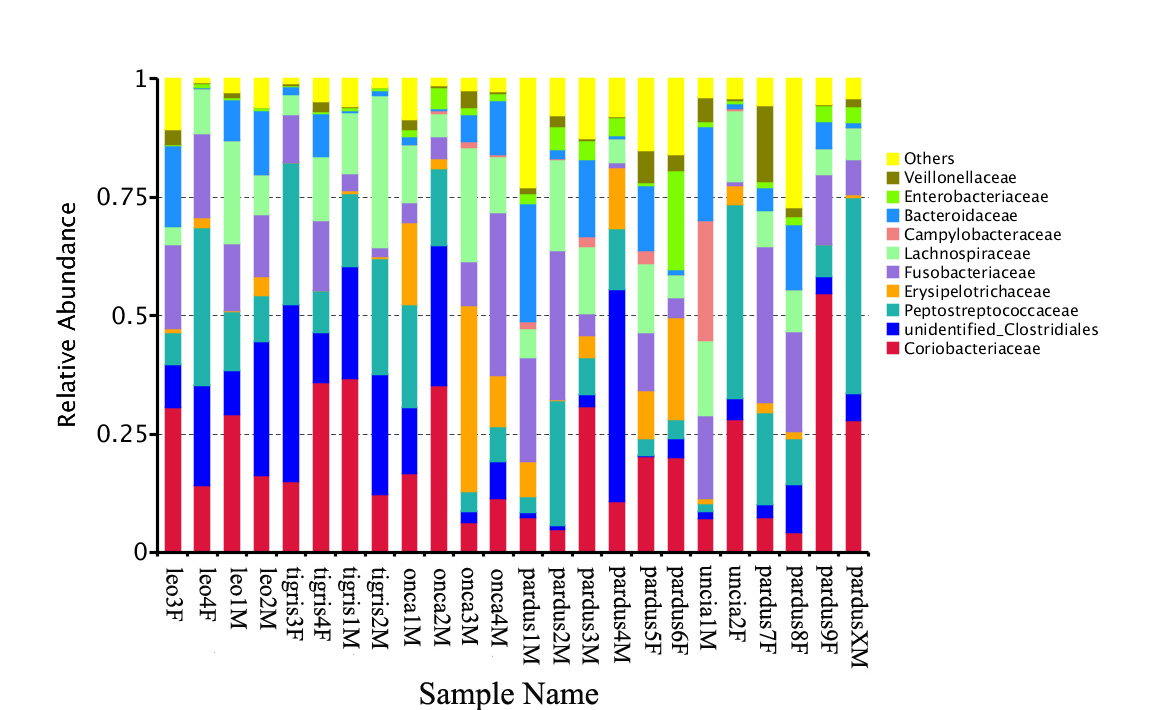

Supplement: Supplementary Figure 3 — The relative abundance of fecal microbes at the family level. [file Image_3.JPEG]

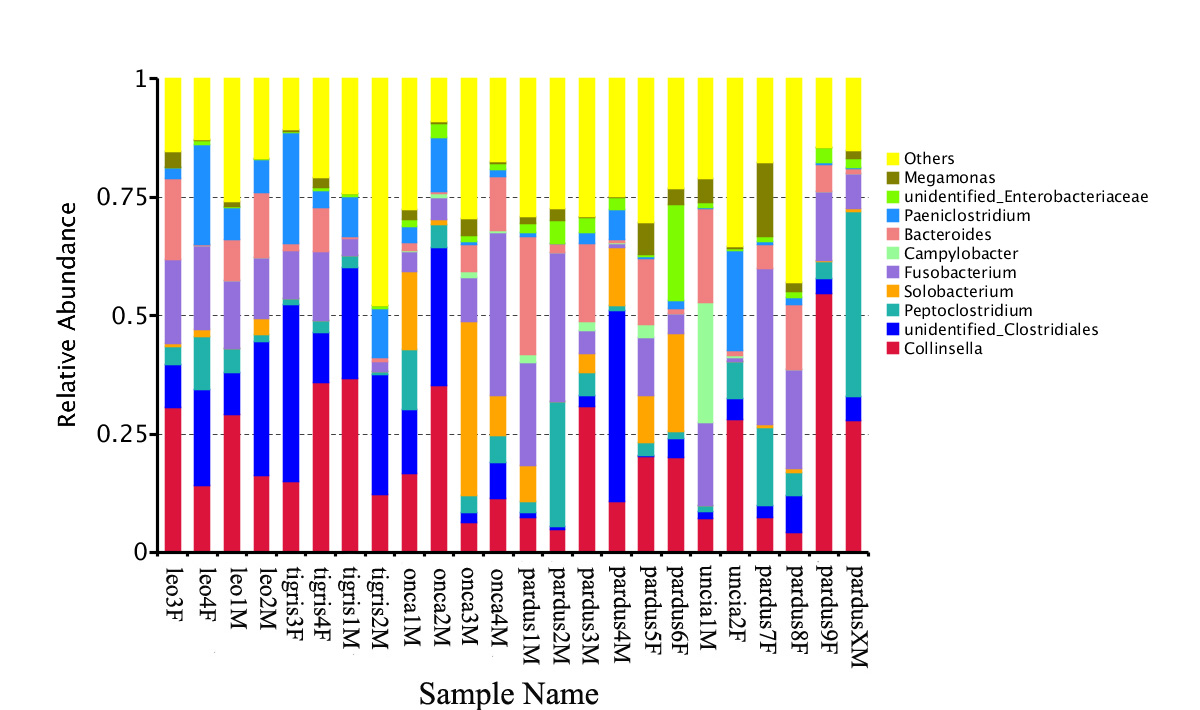

Supplement: Supplementary Figure 4 — The relative abundance of fecal microbes at the genus level. [file Image_4.JPEG]

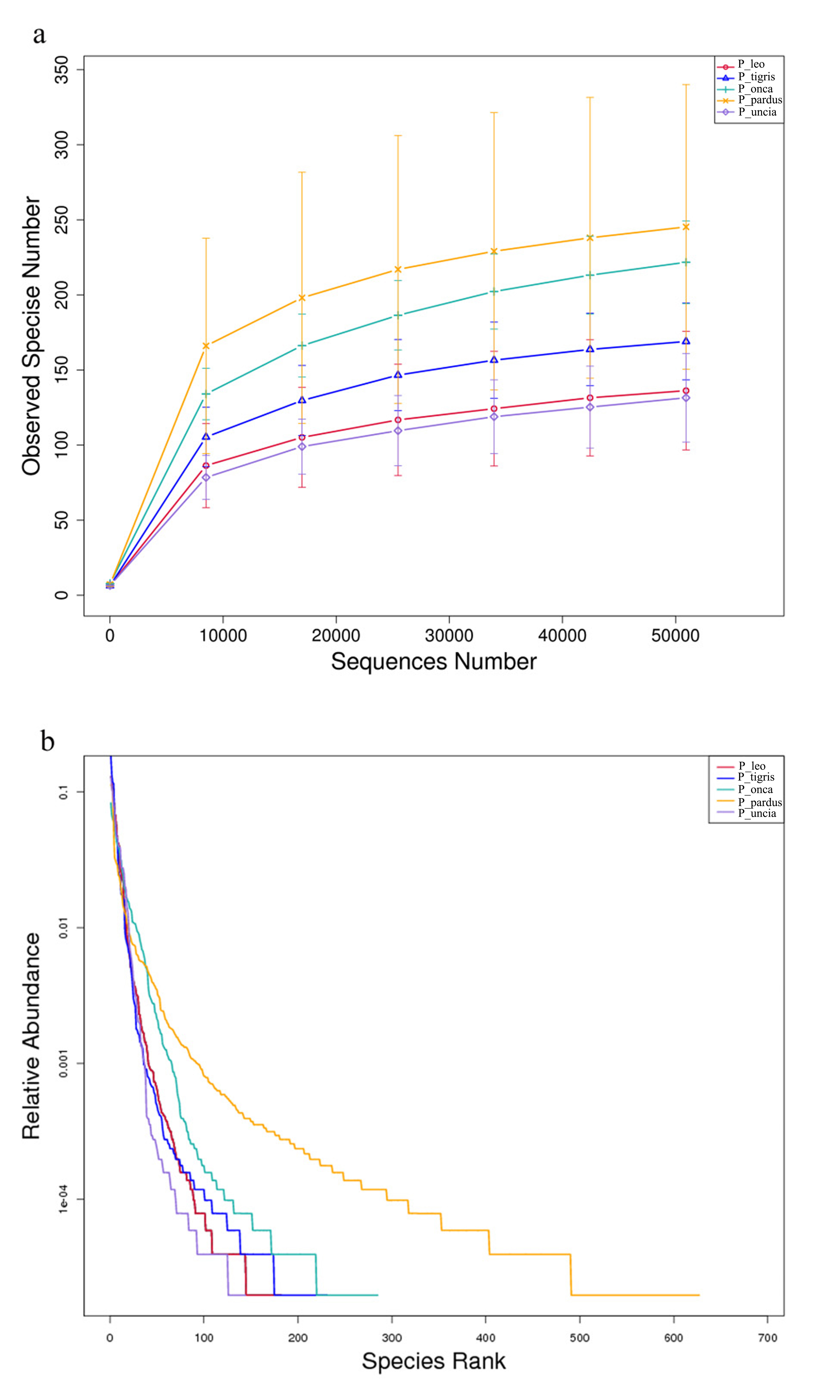

Supplement: Supplementary Figure 5 — (A) Rarefaction curve of the fecal microbiota of species groups. (B) Rank abundance curve of the fecal microbiota of species groups. [file Image_5.JPEG]

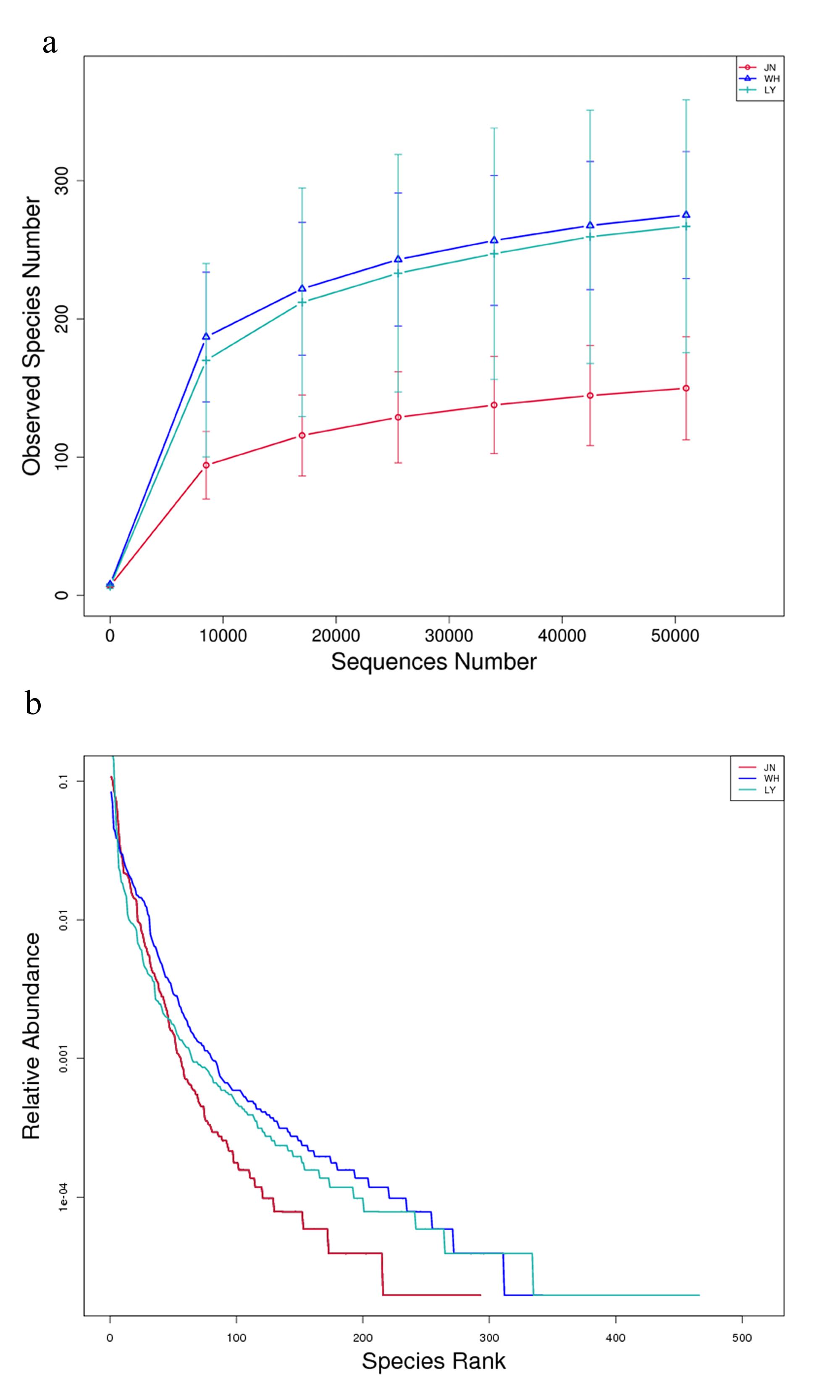

Supplement: Supplementary Figure 6 — (A) Rarefaction curve of the fecal microbiota of sampling location groups. (B) Rank abundance curve of the fecal microbiota of sampling location groups. [file Image_6.JPEG]

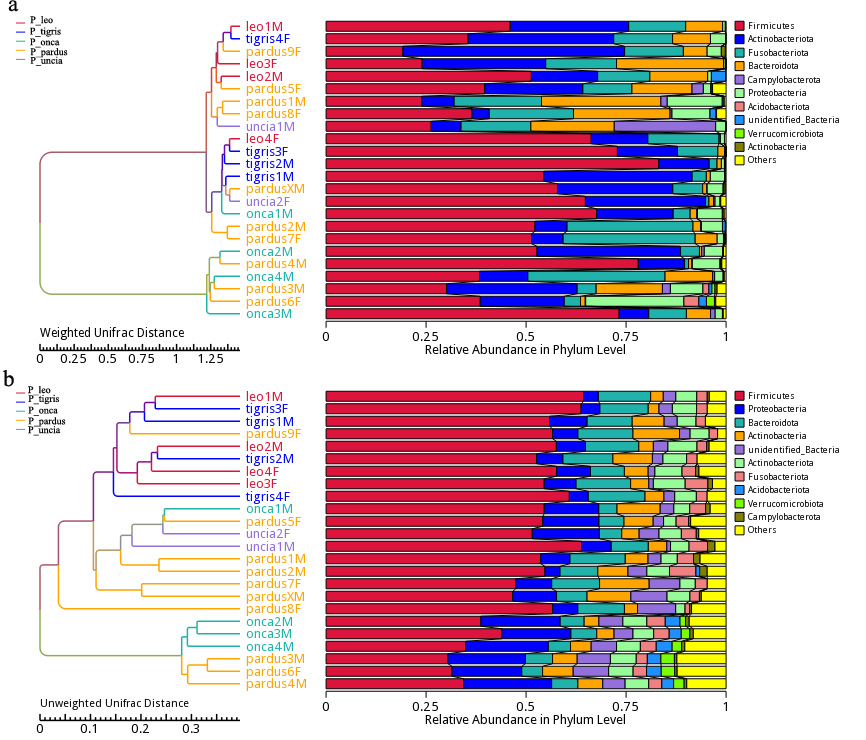

Supplement: Supplementary Figure 7 — The UPGMA clustering analysis of different species is based on weighted (A) and unweighted (B) UniFrac distance. [file Image_7.JPEG]

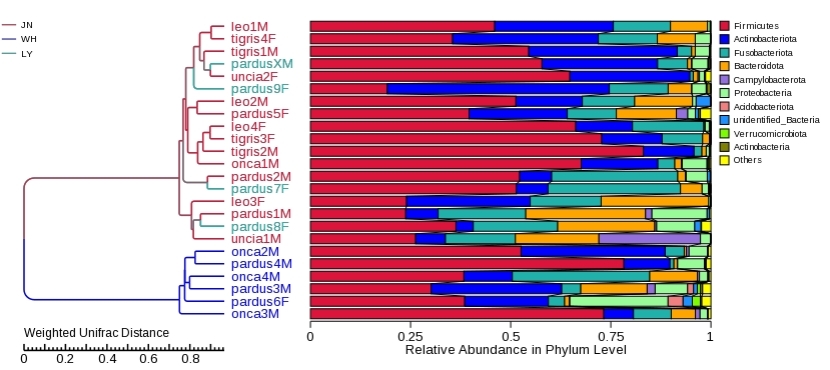

Supplement: Supplementary Figure 8 — The UPGMA clustering analysis of different sampling location groups based on weighted UniFrac distance. [file Image_8.JPEG]

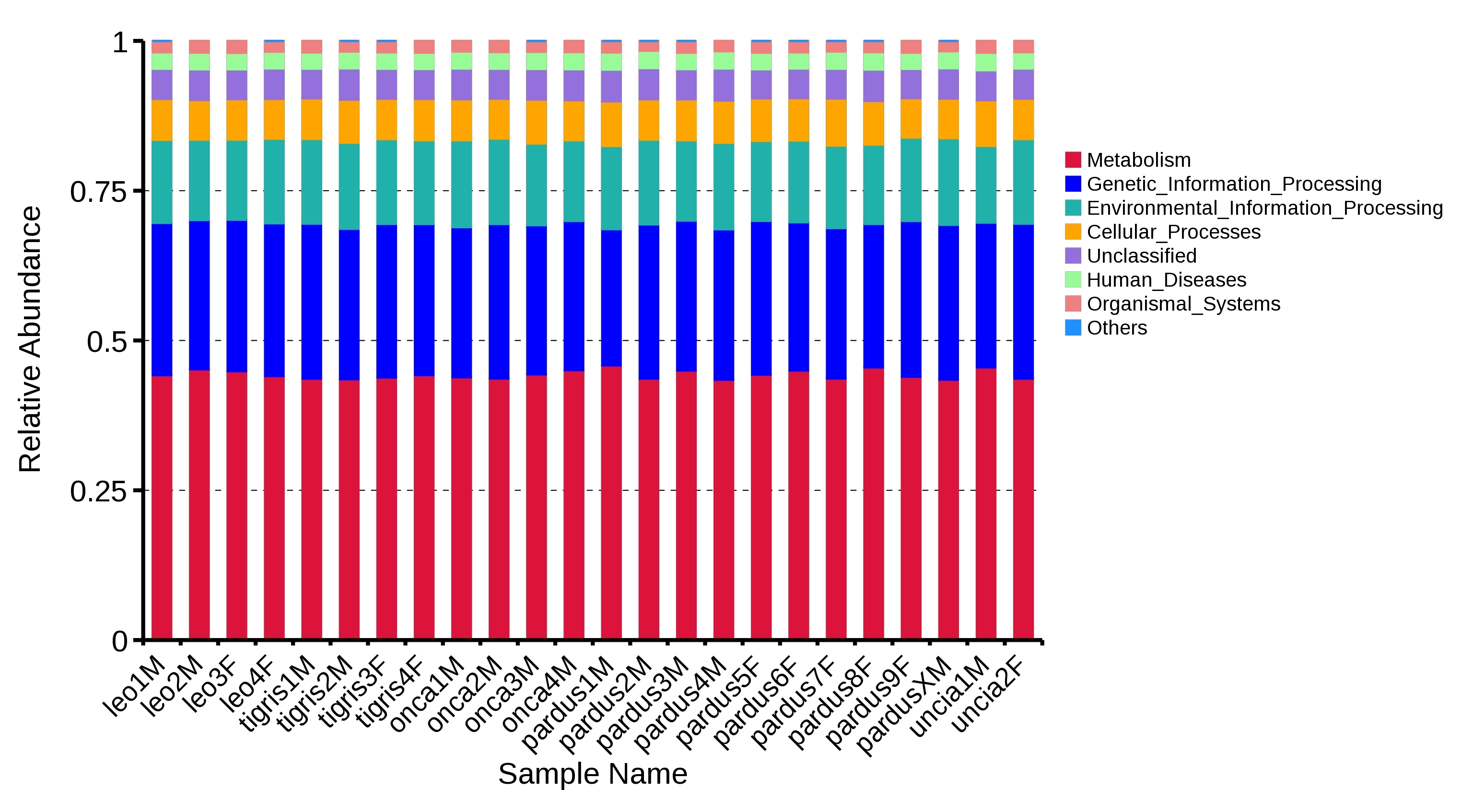

Supplement: Supplementary Figure 9 — Relative functional abundances of fecal microbes annotated at the KEGG level 1. [file Image_9.JPEG]

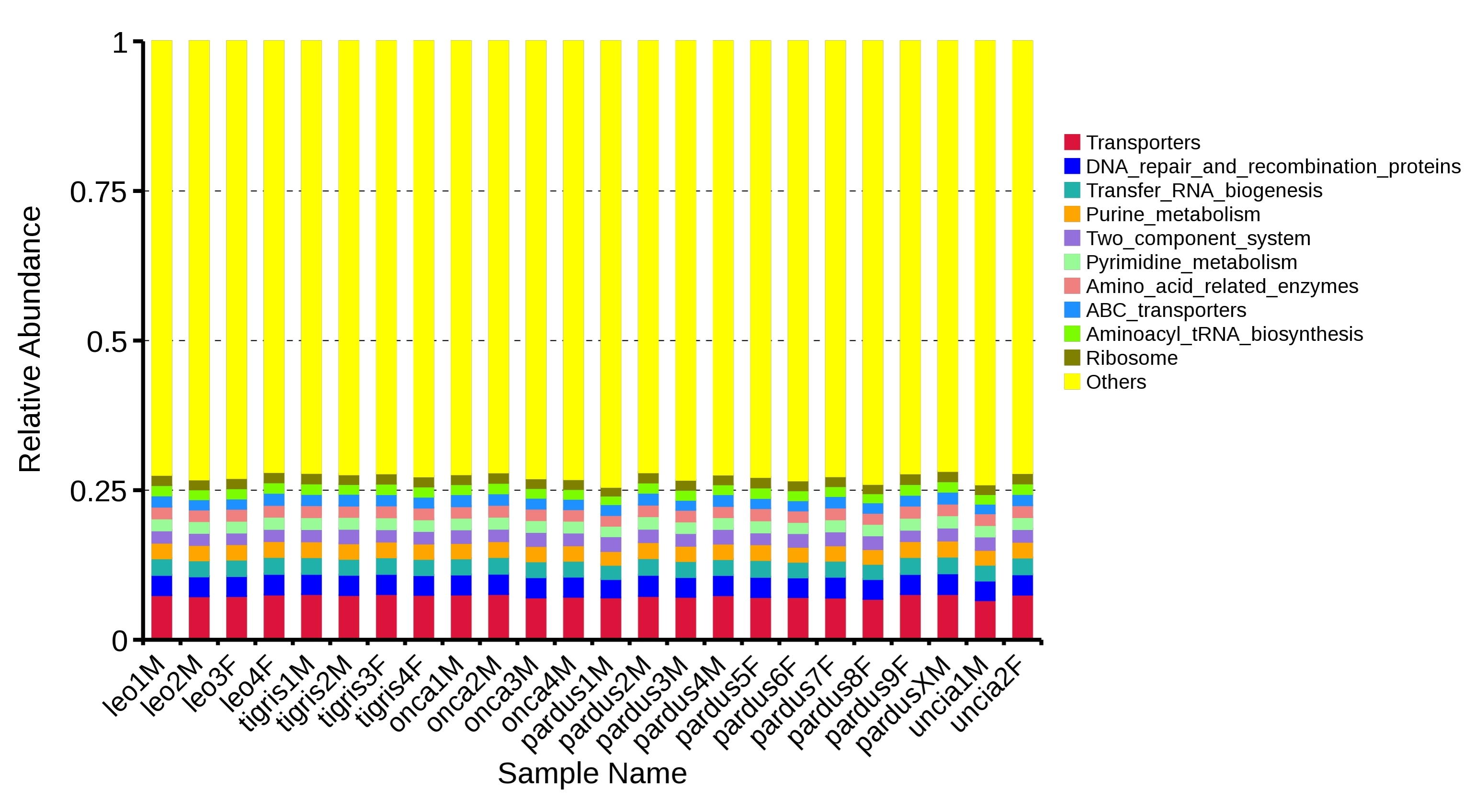

Supplement: Supplementary Figure 10 — Relative functional abundances of fecal microbes annotated at the KEGG level 3. [file Image_10.JPEG]

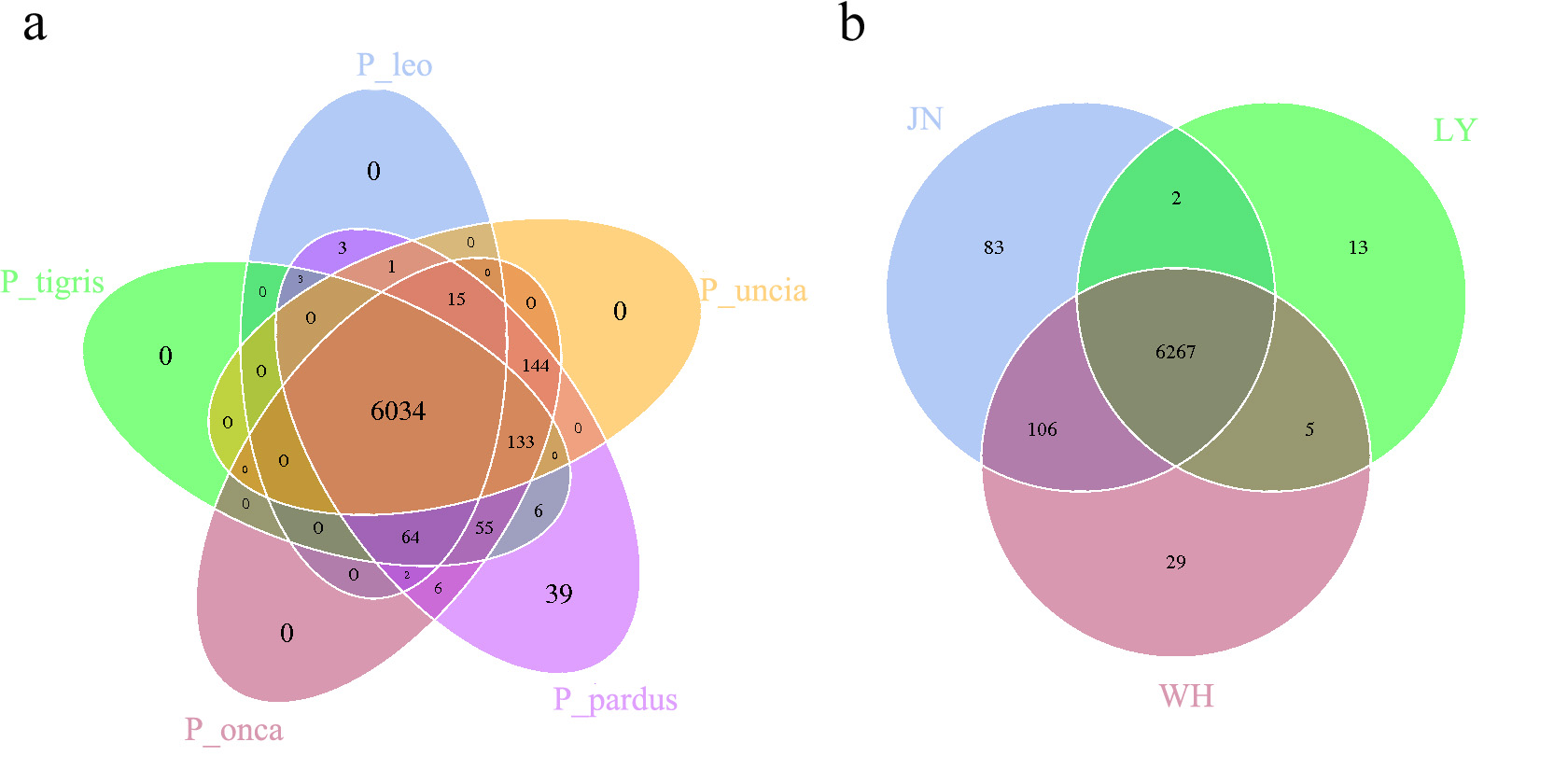

Supplement: Supplementary Figure 11 — A venn diagram shows the abundance of functions shared and unique among different species (A) and different sampling location groups (B). [file Image_11.JPEG]

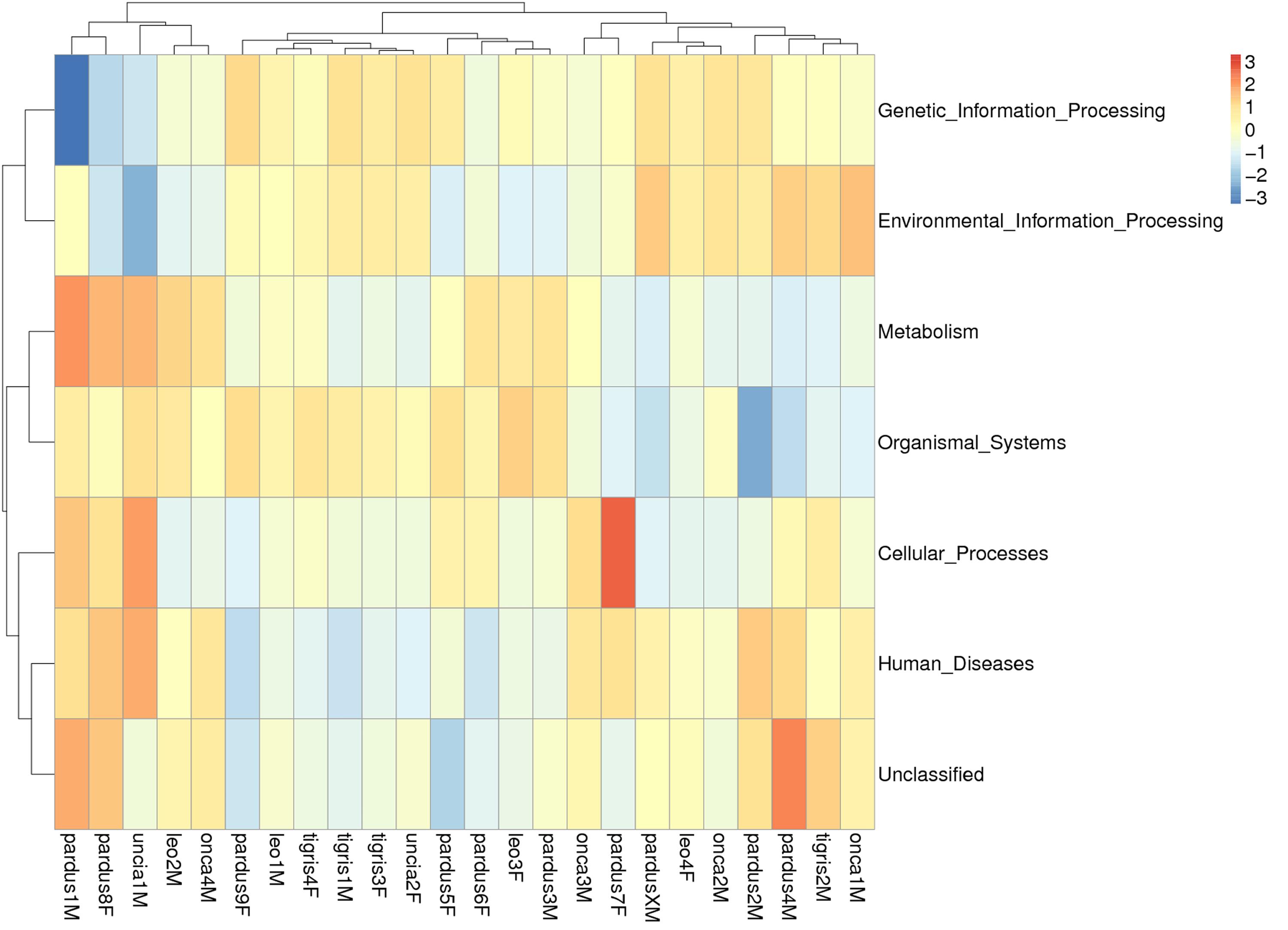

Supplement: Supplementary Figure 12 — The clustering heat map shows the difference in predicted microbial functions among different groups. [file Image_12.JPEG]
