# Supplementary material for: The effect of environment on intestinal microbial diversity of Panthera animals may exceed genetic relationship
Source: Front Microbiol. 2022 Jul 28;13:938900. doi: 10.3389/fmicb.2022.938900 (PMC9366613; doi:10.3389/fmicb.2022.938900)
Supplement: Supplementary Table 1 — ANOSIM analysis based on Bray–Curtis distance (R-value). [file Data_Sheet_1.docx]

| Table S1 Anosim analysis based on Bray Curtis distance (*R-*value). | | | | | | | | |
| --- | --- | --- | --- | --- | --- | --- | --- | --- |
| Group 1 (species difference) | | | | Group 2 (regional differences) | | | | |
| P_pardus – P_onca | | 0.012 | | JN – LY | | 0.159 | | |
| P_tigris – P_onca | | 0.563 | | WH – LY | | 0.917 | | |
| P_tigris – P_pardus | | 0.174 | | WH – JN | | 0.625 | | |
| P_leo – P_onca | | 0.479 | |  | |  | | |
| P_leo – P_pardus | | 0.084 | |  | |  | | |
| P_leo – P_tigris | | 0.063 | |  | |  | | |
| Table S2 MRPP analysis of OTU abundance of different species. | | | | | | | |  |
| Group | A | | Observed delta | | Expected delta | | Significance |  |
| P_onca-P_pardus | 0.013 | | 0.669 | | 0.678 | | 0.191 |  |
| P_leo-P_pardus | 0.062 | | 0.628 | | 0.670 | | 0.005 |  |
| P_leo-P_onca | 0.089 | | 0.562 | | 0.617 | | 0.032 |  |
| P_pardus-P_tigris | 0.095 | | 0.610 | | 0.674 | | 0.003 |  |
| P_onca-P_tigris | 0.137 | | 0.531 | | 0.615 | | 0.026 |  |
| P_leo-P_tigris | 0.021 | | 0.460 | | 0.469 | | 0.240 |  |
| A smaller Observe Delta value indicates a small difference within the group, larger Expect delta values indicate a large difference between the groups. A > 0 indicates that differences between groups are greater than differences within the group, and A < 0 indicates that differences are greater than differences between groups. Significance < 0.05 indicates a significant difference. | | | | | | | |  |

| Table S3 Amova analysis of different groups (*P-*value, *P* < 0.05 indicates significant difference, *P <* 0.01 indicates extremely significant difference) | | |
| --- | --- | --- |
|  | Analysis based on unweighted unifrac distance | Analysis based on weighted unifrac analysis distance |
| P_leo - P_onca - P_pardus - P_tigris - P_uncia | 0.002 | 0.050 |
| JN - LY - WH | 0.001 | 0.001 |

| Table S4 MRPP analysis of OTU abundance of different sampling groups. | | | | |
| --- | --- | --- | --- | --- |
| Group | A | Observed delta | Expected delta | Significance |
| JN-LY | 0.0210 | 0.5934 | 0.6061 | 0.095 |
| JN-WH | 0.0997 | 0.5944 | 0.6603 | 0.001 |
| LY-WH | 0.1381 | 0.5904 | 0.6850 | 0.006 |

| Table S5 Functions with significant different abundance at Level 2 of different species groups | |
| --- | --- |
| Groups | Functions |
| P_leo – P_onca | Signal transduction, Endocrine system, Neurodegenerative diseases |
| P_leo – P_pardus | Carbohydrate metabolism, Signal transduction, Cell motility, Neurodegenerative diseases, Circulatory system |
| P_leo – P_tigris | Glycan biosynthesis and metabolism, Metabolism of cofactors and vitamins,  Lipid metabolism, Transcription, Metabolism, Cell motility, Endocrine system, Substance dependence |
| P_onca – P_tigris | Poorly characterized, Drug resistance, Infectious diseases, Cancers,  Environmental adaptation |
| P_pardus – P_tigris | Membrane transport, Glycan biosynthesis and metabolism, Metabolism of cofactors and vitamins, Transcription, Metabolism, Infectious diseases, Cancers, Digestive system |
